# Meteorological variability and predictive forecasting of atmospheric particulate pollution

**DOI:** 10.1038/s41598-023-41906-8

**Published:** 2024-01-02

**Authors:** Wan Yun Hong

**Affiliations:** Faculty of Integrated Technologies, Universiti Brunei Darussalam, Jalan Tungku Link, Gadong, BE1410 Brunei Darussalam

**Keywords:** Atmospheric dynamics, Projection and prediction

## Abstract

Due to increasingly documented health effects associated with airborne particulate matter (PM), challenges in forecasting and concern about their impact on climate change, extensive research has been conducted to improve understanding of their variability and accurately forecasting them. This study shows that atmospheric PM_10_ concentrations in Brunei-Muara district are influenced by meteorological conditions and they contribute to the warming of the Earth’s atmosphere. PM_10_ predictive forecasting models based on time and meteorological parameters are successfully developed, validated and tested for prediction by multiple linear regression (MLR), random forest (RF), extreme gradient boosting (XGBoost) and artificial neural network (ANN). Incorporation of the previous day’s PM_10_ concentration (PM_10,t-1_) into the models significantly improves the models’ predictive power by 57–92%. The MLR model with PM_10,t-1_ variable shows the greatest capability in capturing the seasonal variability of daily PM_10_ (RMSE = 1.549 μg/m^3^; R^2^ = 0.984). The next day’s PM_10_ can be forecasted more accurately by the RF model with PM_10,t-1_ variable (RMSE = 5.094 μg/m^3^; R^2^ = 0.822) while the next 2 and 3 days’ PM_10_ can be forecasted more accurately by ANN models with PM_10,t-1_ variable (RMSE = 5.107 μg/m^3^; R^2^ = 0.603 and RMSE = 6.657 μg/m^3^; R^2^ = 0.504, respectively).

## Introduction

Air pollution is the world’s largest environmental health risk that accounts for 6.7 million deaths every year with 4.2 million deaths globally in 2019 due to exposure to atmospheric particulate matter that causes cardiovascular and respiratory diseases, and cancers according to recent estimates by the World Health Organisation (WHO)^1^. The greatest atmospheric (outdoor) air pollution-related deaths were in the Southeast Asia region. The impact of air pollution on human health is of growing concern due to increasing exposure to air pollution, with almost all (99%) of the world’s population in 2019 living in areas where the air pollution levels exceed the safe WHO air quality guideline (AQG) limits^2^. There is also concern about the implications of air pollution on the climate and ecosystem around the world^3,4^. Therefore, concerted action is urgently needed to reduce air pollution to protect the populations from health risks and mitigate climate change.

Effective and sustainable air quality management strategies for cleaner air need to be implemented to address the global air pollution emergency. Research relating to air pollution has been receiving remarkable interest due to the urgency of understanding the influence of air pollution and its trends^5^. An air quality forecasting model could be an important tool in providing estimates and future predictions of air pollutant concentrations for policymakers to develop legislation and policies to reduce air pollution as well as to alert the public when air pollution events are expected^6^. Two common methods used in air quality forecasting are statistical modelling and chemical transport modelling, which can be based on meteorological conditions to account for atmospheric dilution and diffusion capacity^5,7^. Statistical models are suitable for describing site-specific associations between air pollutants and meteorological parameters, they are easier, faster and more accurate than the chemical transport models^8^ and no costly emission inventories and computer resources are required^9^. However, statistical models are highly dependent on the time series data and they require a large amount of historical data^5^.

A popular statistical method uses machine learning models^10^. Lasheras et al. (2020) built and analyze different statistical and machine learning PM_10_ forecasting models based on the concentrations of six air pollutants: PM_10_, sulfur dioxide (SO_2_), nitrogen monoxide (NO), nitrogen dioxide (NO_2_), carbon monoxide (CO) and ozone (O_3_) as input variables^11^. For instance, deep learning models (a subset of machine learning) such as artificial neural networks (ANNs) tend to have higher accuracy than statistical models but they are unstable and have a high dependence on data^5^. On the other hand, the chemical transport models explicitly describe all major physico-chemical and meteorological processes associated with air pollution^12^. The drawbacks of chemical transport models are inaccuracies in describing the physico-chemical processes due to inadequate information on pollutant sources and they are not able to accurately predict extreme events, the spatio-temporal variation of air pollutants and time series for short and medium ranges^9^.

The atmospheric air quality in Brunei Darussalam, a Southeast Asian country, is usually considered clean despite being seasonally affected by the transboundary smoke haze, in which the atmosphere contains high concentrations of particulate matter (PM), especially PM equal to or smaller than 10 µm in diameter (PM_10_) that can penetrate deep into the lungs. The highest daily PM_10_ concentration observed across Brunei Darussalam was 100.90 μg/m^3^ (moderate air quality) in September 2019 during the south-west (SW) monsoon season, which was caused by the transboundary smoke haze from hotspots in the Borneo region^13^. Although various approaches have been proposed for air pollution forecasting, statistical/empirical models for predicting the concentrations of atmospheric PM in Brunei-Muara district from time and meteorological inputs are yet to be developed.

For this reason, the present study aims to analyze the temporal variations and associations of PM_10_ concentrations and meteorological parameters, and develop predictive forecasting models for atmospheric PM_10_ concentration in Brunei-Muara district that accounts for changes in meteorology over time. The objectives of the study are:

1. to determine the correlation between PM_10_ and the individual meteorological parameters (such as temperature, wind speed, wind direction and total rainfall) in different monsoon seasons as well as the combined effects of different rain and wind conditions on PM_10_ to examine their contribution to atmospheric PM_10_ pollution;
2. to build PM_10_ predictive forecasting models using four modelling approaches (such as multiple linear regression (MLR), random forest (RF), extreme gradient boosting (XGBoost) and artificial neural network (ANN)) from the available PM_10_ concentration and meteorological data;
3. to add the previous 1, 2 or 3 days’ PM_10_ concentration to the model to enhance the model’s predictive power in explaining variability^14^;
4. to validate and test the models for predicting and forecasting the next 1, 2 or 3 days’ PM_10_ concentration of the studied area; and
5. to evaluate the models’ performances in explaining the daily variability of PM_10_ concentration.

The presented results will provide a better understanding of the effect of meteorological parameters on atmospheric PM_10_ in Brunei-Muara district. To the best of the author’s knowledge, this study is the first to explore and quantify the combined effects of different rain and wind conditions on PM_10_ concentrations in Brunei-Muara district, which can be helpful to policymakers when developing air pollution mitigation measures or assessing the effectiveness of those measures for the studied area. The study also intends to demonstrate the significant potential of the proposed PM_10_ predictive forecasting models in producing more accurate PM_10_ predictions that can provide early information against unhealthy atmospheric PM_10_ concentrations to the local community so that the necessary precautionary measures can be taken to minimize exposure to air pollution to protect public health.

### Study area and data

**Figure 1:**
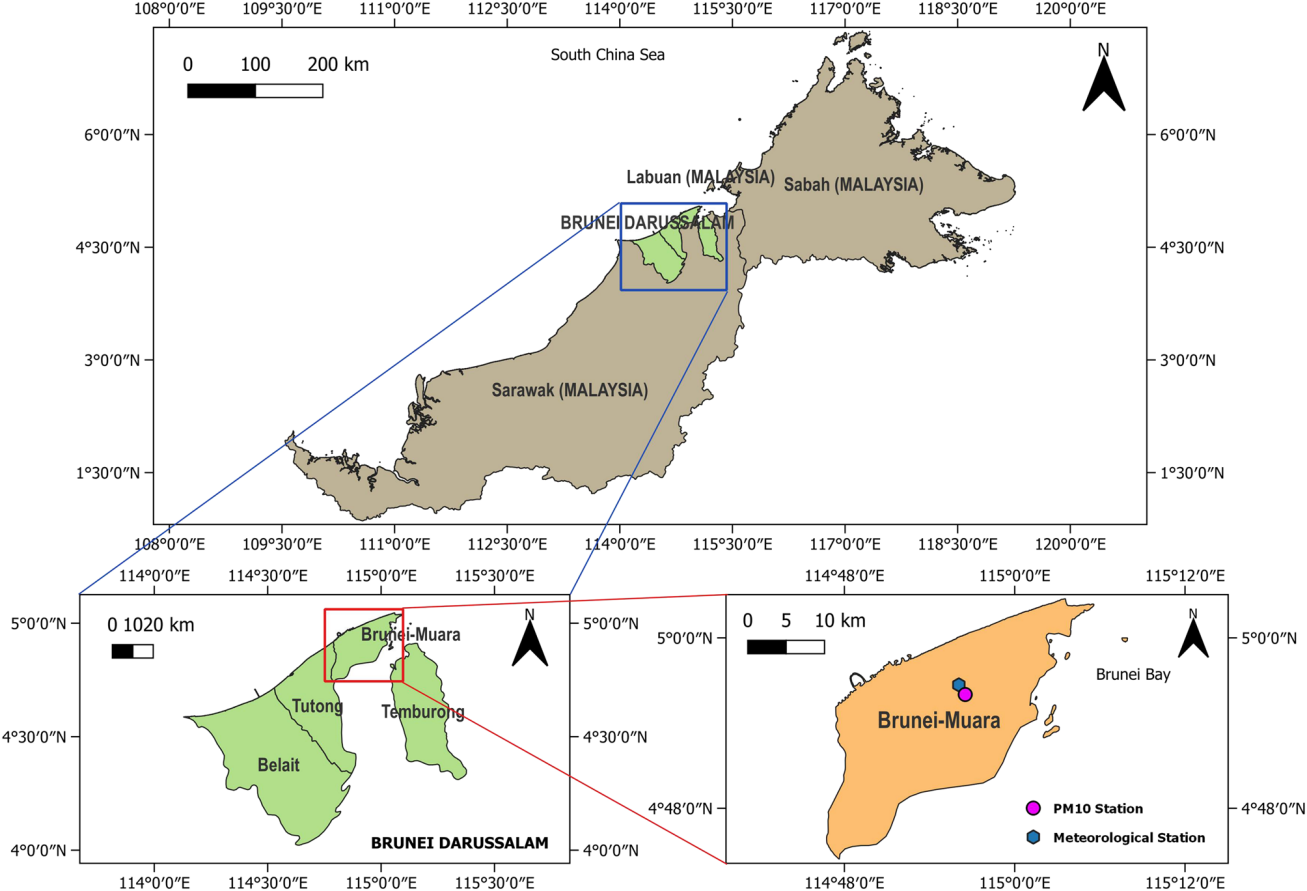
Locations of PM_10_ and meteorological monitoring stations in Brunei-Muara district, Brunei Darussalam.

Brunei-Muara district is located in the northeast of Brunei Darussalam, bordering the South China Sea and Labuan (Malaysia) to the north, Brunei Bay to the east, Limbang, Sarawak (Malaysia) to the south and the Bruneian district of Tutong to the west^15^ (Fig. 1). It is the most populated district in Brunei Darussalam, with 318,530 people (as of 2021), containing about 72% of the country’s population although it has the smallest area (570 km^2^) among the four districts of Brunei Darussalam^15,16^. Brunei-Muara is where the country’s capital (Bandar Seri Begawan), the seat of government ministries and departmental headquarters, and the center of commercial activities are located^15^. It also houses the country’s only international airport (Brunei International Airport) and main deep-water port (Muara Port).

Daily average PM_10_ concentrations (μg/m^3^) and meteorological data measured in Brunei-Muara district from 2009 to 2019 (11 years) were obtained from the Department of Environment, Parks and Recreation (JASTRe), Ministry of Development, Brunei Darussalam and the Brunei Darussalam Meteorological Department (BDMD), Ministry of Transport and Infocommunications, Brunei Darussalam, respectively. The meteorological parameters include the daily average temperature (°C), wind speed (m/s) and wind direction (°), and daily total rainfall (mm). The time parameters are the observations’ day, month and year. The data are classified into four monsoon seasons, namely: north-east (NE) monsoon (from December to March), inter-monsoon 1 (from April to May), south-west (SW) monsoon (from June to September) and inter-monsoon 2 (from October to November).

## Methods

This study employed XLSTAT software for statistical analysis and modelling. The statistical analysis includes descriptive statistics (such as minimum, maximum, mean and standard deviation) of daily average PM_10_ concentrations and meteorological parameters during different seasons in Brunei-Muara district from 2009 to 2019. To explore the impact of meteorological parameters on PM_10_ pollution in different seasons, Pearson correlation tests were performed between daily average concentrations of PM_10_ ($y$-variable) and daily average temperature, wind speed and wind direction, and daily total rainfall ($x$-variables) that were recorded from 2009 to 2019, with the seasons as the subsamples. The Pearson correlation coefficient $r$ was calculated using:

$$r = \frac{{n\left( {\sum xy} \right) - \left( {\sum x} \right)\left( {\sum y} \right)}}{{\sqrt {\left[ {n\sum x^{2} - \left( {\sum x} \right)^{2} } \right]\left[ {n\sum y^{2} - \left( {\sum y} \right)^{2} } \right]} }}, - 1 < r < 1$$

where $n$ is the number of observations, $x$ is the value of the $x$-variable in the sample and $y$ is the value of the $y$-variable in the sample. A Pearson correlation coefficient represents the degree of the linear correlation between two variables. The correlations were also tested with a significance level of 5%. The computed coefficients of determination R^2^ correspond to the squared of the Pearson correlation coefficients, which measure the strength of the correlation.

Data from 2009 to 2018 (10 years) were first modelled using the MLR approach to describe the variance of the time and meteorological parameters on the daily variation of PM_10_ concentrations for seven scenarios. For Scenario 1/Baseline (i.e., PM_10_ model with time and meteorological parameters only), the explanatory variables were the time parameters (that include the observations’ day, month and year) and the meteorological parameters (that include the daily temperature, wind speed, wind direction and total rainfall). For Scenarios 2 to 4 (i.e., PM_10_ models with the previous day’s PM_10_, the average of the previous 2 days’ PM_10_ and the average of the previous 3 days’ PM_10_; which are denoted by PM_10,t-1_, PM_10,t-2_ and PM_10,t-3_, respectively), the explanatory variables were all the time and meteorological parameters and the PM_10,t-1_, PM_10,t-2_ or PM_10,t-3_ variable. For Scenarios 5 to 7 (i.e., PM_10_ models for the next 1 day, 2 days and 3 days; which are denoted by PM_10,t+1_, PM_10,t+2_ and PM_10,t+3_, respectively), the explanatory variables were all the time and meteorological parameters, the corresponding PM_10_ concentrations and the PM_10,t-1_ variable. Variables that might be either constant or too correlated with other variables used in the model were not taken into account by the model and the model’s tolerance value was 0.0001. The interactions in the model were set at 3 and the best model was selected based on the lowest mean square errors (MSE). The minimum variables for all MLR models were set at 2 and the maximum variables were set at 7 for Scenario 1/Baseline, 8 for Scenarios 2 to 4, and 9 for Scenarios 5 to 7. In this study, 90% of the data were used for model learning and 10% of the data were randomly selected for model validation. Once the best model was built, it was tested to make predictions on the 2019 data (1 year).

Then, the RF approach was applied to build more efficient predictive regression models for daily PM_10_ concentrations by generating several predictors and then combining their respective predictions. Data from 2009 to 2018 (10 years) were used to develop random forest PM_10_ models for the seven scenarios. The explanatory variables used in the RF models for Scenarios 1 to 7 were the same as those used in the MLR method. For the forest parameters, the sampling method was random with replacement and the forest type was bagging with 90% of the data used to generate the trees. The number of trees in the forest was set at 300 and the maximum allowable construction time of all trees in the forest was set at 300 s with a convergence of the machine learning algorithm set at every 100 trees. For the tree parameters, the minimum node size was 7 for Scenario 1, 8 for Scenarios 2 to 4) and 9 for Scenarios 5 to 7, respectively). The minimum son size was set at 2 and the maximum tree depth was set at 20 with a complexity parameter value of 0.0001 for all the models. All the random forest PM_10_ models were validated with 10% of the data, which were randomly selected, and tested to make predictions on the 2019 data (1 year).

Next, an XGBoost approach was employed to build combined boosted ensemble regression models for daily PM_10_ concentrations prediction based on the available data from 2009 to 2018 (10 years) for the seven scenarios. The explanatory variables used in the XGBoost models for Scenarios 1 to 7 were the same as those used in the MLR method. The maximum number of iterations of the model was 50 and the learning rate was 0.3 with zero minimum loss reduction. The objective/loss function was quadratic and the metric of the loss function was the root mean square error (RMSE). For the tree parameters, the minimum son size was 2 and the maximum tree depth was 6. When the PM_10_ models for Scenarios 1 to were built, they were validated with 10% of the data (selected at random) and then tested to make predictions on the 2019 data (1 year).

Lastly, an ANN approach was employed to build more complex and efficient predictive regression models for daily PM_10_ concentrations for the seven scenarios. The ANN model consists of an input layer (explanatory variables for Scenarios 1 to 7), two hidden layers and an output layer (PM_10_ variable for Scenario 1 to 4, PM_10,t+1_ variable for Scenario 5, PM_10,t+2_ variable for Scenario 6 and PM_10,t+3_ variable for Scenario 7) with a group of interconnected nodes (artificial neurons). The explanatory variables used in the ANN models for Scenarios 1 to 7 were the same as those used in the MLR method. The neuralnet function in XLSTAT-R was used, which calls the neuralnet function from the neuralnet package in R developed by Stefan Fritsch and Frauke Guenther (2022)^17^. Data from 2009 to 2019 (11 years) were rescaled and randomly split into a training sample (80% of the data), a validation sample (10% of the data) and a test sample (10% of the data). The number of neurons in the hidden layers was 2,2 (that corresponds to 2 neurons in the first hidden layer and 2 neurons in the second hidden layer) for Scenario 1, 3,2 for Scenarios 2 to 4, and 4,2 for Scenarios 5 to 7. The threshold value was 0.01 and the maximum steps were 100,000. The algorithm RProp + was chosen, which refers to resilient backpropagation with weight backtracking. The error function was squared errors and the activation function was logistic with linear output.

The models’ error and accuracy between the predicted and observed values for Scenarios 1 to 7 were evaluated by several performance metrics, which include the RMSE, R^2^, mean absolute percentage error (MAPE), Willmott’s index of agreement (WIA) and Legates and McCabe index (LCI). The best model was chosen when it has a minimal error (i.e., RMSE close to 0 and/or MAPE close to 0%) and it has high accuracy (i.e., R^2^, WIA and/or LCI close to 1).

## Results and discussion

**Figure 2:**
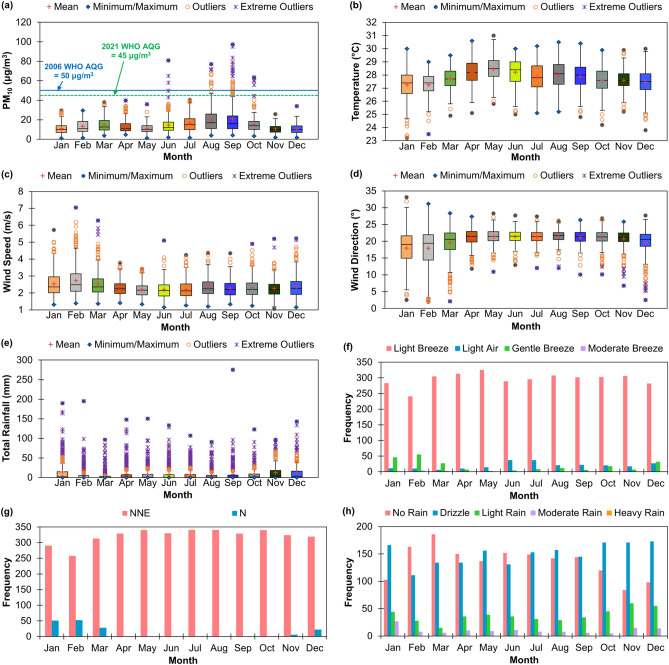
Monthly variations of PM_10_ concentrations (**a**), temperature (**b**), wind speed (**c**), wind direction (**d**) and total rainfall (**e**), and monthly frequencies of the classified wind types (**f**), wind (cardinal) directions (**g**) and rainfall types (**h**) in Brunei-Muara district from 2009 to 2019.

Table 1 summarizes the seasonal characteristics of PM_10_ pollution and meteorological conditions in Brunei-Muara district from 2009 to 2019. In this time period, only 1.1% of the daily average PM_10_ concentrations were greater than the 2006 WHO Global AQG limit for daily average PM_10_ concentration (i.e., 50 μg/m^3^)^18^ and only 1.4% of the daily average PM_10_ concentrations were greater than the 2021 WHO Global AQG limit for daily average PM_10_ concentration (i.e., 45 μg/m^3^)^19^, mostly during the SW monsoon season in June, August and September, and occasionally continued during the inter-monsoon 2 season in October (Fig. 2a). The maximum daily average concentration of PM_10_ observed in Brunei-Muara was 97.4 μg/m^3^ in September 2019, with a seasonal mean concentration of PM_10_ of 17.9 μg/m^3^ and a standard deviation of 12.2 μg/m^3^ during the SW monsoon season (Table 1).

**Table 1:** Statistical summary of seasonal PM_10_ pollution and meteorological conditions in Brunei-Muara district from 2009 to 2019.

Season & Period	Statistic	PM_10_ (µg/m^3^)	Temperature (°C)	Wind speed (m/s)	Wind direction (°)	Total rainfall (mm)
NE Monsoon (Dec–Mar)	Minimum	1.0	23.2	1.2	2.0	0
	Maximum	38.0	30.0	7.1	33.1	195.1
	Mean	12.3	27.4	2.5	18.8	9.9
	Standard Deviation	6.2	1.0	0.8	5.0	21.1
Inter-Monsoon 1 (Apr–May)	Minimum	1.1	25.1	1.3	10.9	0
	Maximum	39.7	31.0	3.8	28.3	150.2
	Mean	12.1	28.3	2.2	21.3	8.6
	Standard Deviation	5.8	0.9	0.4	2.4	18.6
SW Monsoon (Jun–Sep)	Minimum	1.5	24.8	1.2	10.1	0
	Maximum	97.4	30.5	5.1	27.7	275.0
	Mean	17.9	28.0	2.3	21.3	7.6
	Standard Deviation	12.2	1.0	0.5	2.0	17.3
Inter-Monsoon 2 (Oct–Nov)	Minimum	2.0	24.2	1.1	6.8	0
	Maximum	63.5	29.9	5.2	26.9	122.9
	Mean	12.8	27.6	2.3	21.0	10.3
	Standard Deviation	6.9	0.9	0.5	2.5	17.0

Brunei-Muara district has a tropical climate and it is usually very warm and wet throughout the year. From 2009 to 2019, the daily average temperatures ranged between 23.2 °C and 31.0 °C, with the hottest day recorded in May 2014 (Table 1 and Fig. 2b). The daily wind speed in Brunei-Muara can be as low as 1.1 m/s (as recorded during the Inter-monsoon 2 season in November 2017) and it can reach as high as 7.1 m/s (as recorded during the NE monsoon season in February 2016) (Fig. 2c). The wind types experienced in Brunei-Muara from 2009 to 2019 were either light air (0.3–1.5 m/s), light breeze (1.6–3.3 m/s), gentle breeze (3.4–5.4 m/s) or moderate breeze (5.5–7.9 m/s) (Fig. 2f) based on the wind classifications described by the World Meteorological Organization (WMO)^20^. Usually, the wind blows as a light breeze throughout the year (Fig. 2f), with monthly mean wind speeds ranging from 2.20 m/s to 2.74 m/s (Fig. 2c). The prevailing winds were from the north-northeast (NNE) direction throughout the year (Fig. 2g), with mean wind degree directions between 18.8° and 21.3° (Fig. 2d).

The highest daily total rainfall was 275.0 mm in September 2019 (Table 1 and Fig. 2e). The rainfall types that occurred in Brunei-Muara from 2009 to 2019 were either no rain (0 mm/day), drizzle (0.1–19.9 mm/day), light rain (20–59.9 mm/day), moderate rain (60.0–239.9 mm/day) or heavy rain (240.0–1,199.9 mm/day) (Fig. 2h) according to the rainfall classifications recommended by the WMO^20^. Drizzle was the most frequent type of rainfall (44.9% of the total observations) that often occurs during the NE monsoon (December to March) and both inter-monsoon (April to May and October to November) seasons. The dry season usually occurs during the SW monsoon season (June to September), with 43.9% of the observations in this season without any rain.

The Pearson correlation coefficients $r$ between PM_10_ concentrations and the selected meteorological parameters for Brunei-Muara district from 2009 to 2019 were computed for different monsoon seasons (Table 2). PM_10_ was positively and moderately correlated (0.30 $<r<$ 0.49) with temperature during NE monsoon, inter-monsoon 1 and SW monsoon seasons (December to September), and it is positively and weakly correlated ($r<$ 0.29) during the inter-monsoon 2 season (October to November). This implies that PM_10_ absorbs sunlight and warms the Earth’s atmosphere^21^.

**Table 2:** Pearson correlation coefficients between PM_10_ concentrations and meteorological parameters during different monsoon seasons in Brunei-Muara district from 2009 to 2019.

Season & Period	Variable	PM_10_ (µg/m^3^)	Temperature (°C)	Wind Speed (m/s)	Wind Direction (°)	Total Rainfall (mm)
NE Monsoon (Dec–Mar)	PM_10_ (µg/m^3^)	1	0.376*	0.078*	− 0.128*	− 0.195*
	Temperature (°C)		1	0.223*	− 0.193*	− 0.410*
	Wind Speed (m/s)			1	− 0.652*	− 0.037
	Wind Direction (°)				1	0.053
	Total Rainfall (mm)					1
Inter-Monsoon 1 (Apr–May)	PM_10_ (µg/m^3^)	1	0.328*	− 0.257*	0.163*	− 0.083*
	Temperature (°C)		1	− 0.163*	0.172*	− 0.313*
	Wind Speed (m/s)			1	− 0.232*	0.134*
	Wind Direction (°)				1	− 0.044
	Total Rainfall (mm)					1
SW Monsoon (Jun–Sep)	PM_10_ (µg/m^3^)	1	0.415*	− 0.241*	0.149*	− 0.195*
	Temperature (°C)		1	− 0.287*	0.358*	− 0.388*
	Wind Speed (m/s)			1	− 0.204*	0.172*
	Wind Direction (°)				1	− 0.141*
	Total Rainfall (mm)					1
Inter-Monsoon 2 (Oct–Nov)	PM_10_ (µg/m^3^)	1	0.211*	− 0.209*	0.089*	− 0.085*
	Temperature (°C)		1	− 0.196*	0.049	− 0.298*
	Wind Speed (m/s)			1	− 0.187*	0.143*
	Wind Direction (°)				1	− 0.022
	Total Rainfall (mm)					1

*Coefficient is significant at a significance level α = 0.05.

Between PM_10_ and wind speed, a weak positive correlation ($r<$ 0.29) was observed during the NE monsoon season (December to March) and weak negative correlations ($r<-$ 0.29) were observed during SW monsoon and both inter-monsoon seasons (April to November). On the other hand, a weak negative correlation was observed between PM_10_ and wind direction during NE monsoon, and weak positive correlations were observed during SW monsoon and both inter-monsoon seasons. This implies that the atmospheric PM_10_ concentrations can be diluted and diffused by particle dispersion and/or transported to a greater height or to a nearby area when the wind speed increases^22,23^ and blows from the NNE direction, generally during SW monsoon and both inter-monsoon seasons (April to November). However, during the NE monsoon season (December to March), more PM_10_ in the area can be blown away at reduced wind speed when the wind blows from the NNE and N directions.

**Figure 3:**
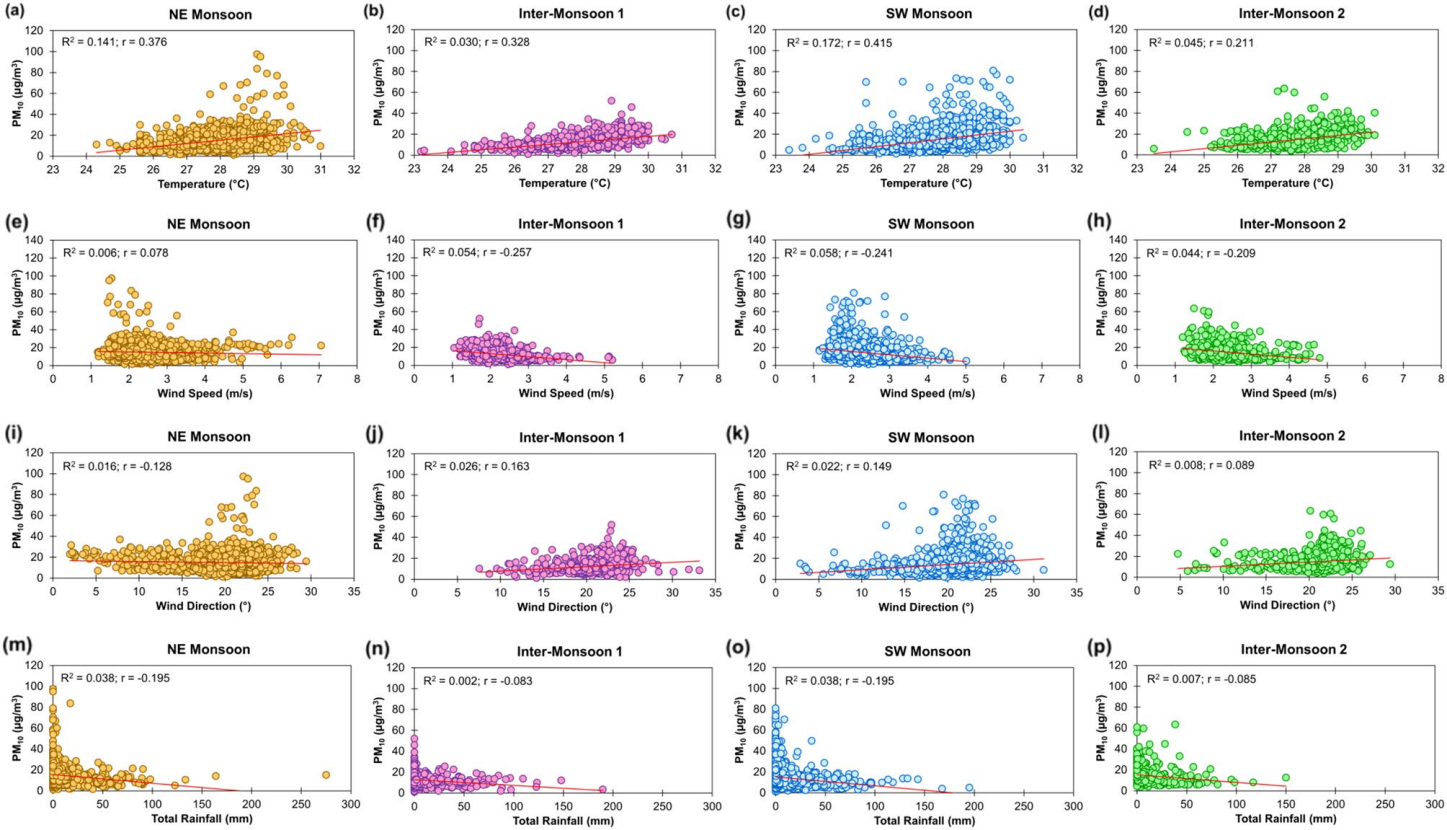
Scatter plots of daily PM_10_ concentrations against temperature (**a–d**), wind speed (**e–h**), wind direction (**i–l**) and total rainfall (**m–p**) during different monsoon seasons in Brunei-Muara district from 2009 to 2019. R^2^ is the coefficient of determination that corresponds to the squared of the Pearson correlation coefficient $r$.

The correlation between PM_10_ and total rainfall was negative and weak in all the seasons, indicating that more PM_10_ in the atmosphere can be washed away when the rain gets heavier^24^. All the coefficients between PM_10_ and the selected meteorological parameters, and most of the coefficients between the selected meteorological parameters were statistically significant (p-values $<$ 0.0001) at a 0.05 significant level (values with an asterisk * in Table 2). The associations between PM_10_ and each of the selected meteorological parameters during different monsoon seasons are illustrated in Fig. 3.

**Figure 4:**
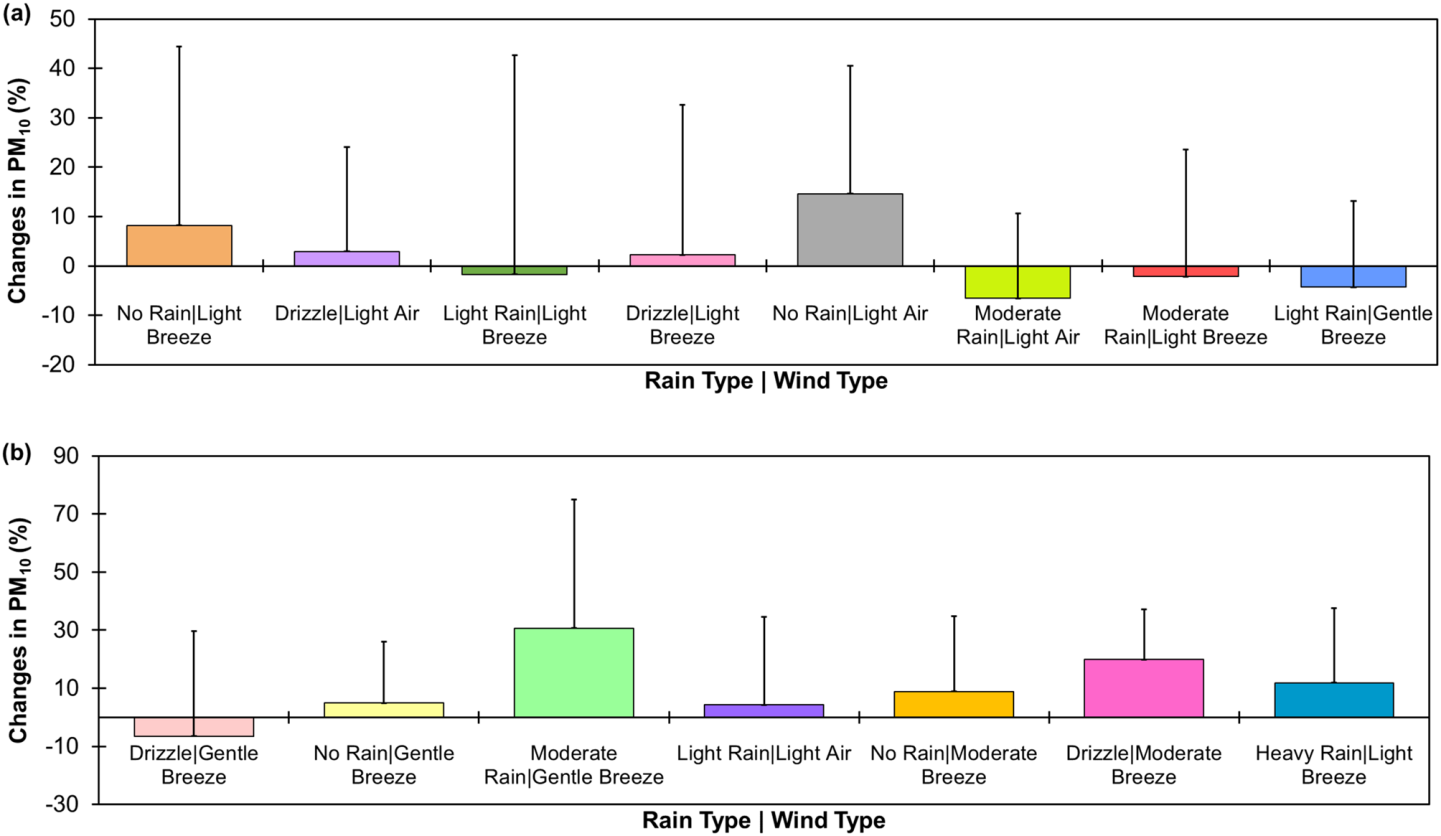
Combined effects of different rain and wind conditions on mean daily PM_10_ concentrations for Brunei-Muara district from 2009 to 2019 (**a–b**).

Figure 4 shows the changes in mean daily PM_10_ concentration (from the previous day) for different rain and wind conditions in Brunei-Muara district from 2009 to 2019. When there was no rain in the day, the mean daily PM_10_ concentration was increased by only 5% (minimum) during a gentle breeze and by 15% (maximum) during light air (Fig. 4a), suggesting that PM_10_ in the atmosphere can be blown away as the wind speed increases. A drizzling day with a gentle breeze can reduce the mean daily PM_10_ concentration by only 6% (Fig. 4b), about 2% more than those during light and moderate rain with a light breeze (Fig. 4a). When the wind speed was increased from light breeze to gentle breeze during light rain, the mean daily PM_10_ concentration was reduced 2.5 times more (i.e., from 1.7% to 4.3%) (Fig. 4a). From 2009 to 2019, moderate rain with light air in Brunei-Muara district reduced the mean daily PM_10_ concentrations by 7% from the previous day’s PM_10_ concentration, the highest among the different rain and wind conditions observed in the area (Fig. 4a).

Four modelling approaches (that include MLR, RF, XGBoost and ANN) were trained, validated and tested for predicting daily PM_10_ concentrations in Brunei-Muara district in seven scenarios. The first/baseline scenario was PM_10_ predictive models with time and meteorological parameters only. The models’ performances are presented in Table 3, which showed very low but acceptable accuracy with a good agreement between the predicted and observed daily PM_10_ concentrations, in which the best results were exhibited by XGBoost during models’ training (RMSE = 2.739 µg/m^3^, R^2^ = 0.898, MAPE = 16.67%, WIA = 0.844 and LCI = 0.687) and validation (RMSE = 4.645 µg/m^3^, R^2^ = 0.593, MAPE = 27.97%, WIA = 0.706 and LCI = 0.411), and ANN during model testing (RMSE = 7.612 µg/m^3^, R^2^ = 0.243, MAPE = 40.20%, WIA = 0.602 and LCI = 0.204). This indicates that some daily variability of PM_10_ in the studied area is not captured by the model. For Scenario 1, the most important variable in predicting PM_10_ concentration is the year (with the highest relative contribution of about 30% to the XGBoost model and 29% to the RF model among the variables), suggesting that there is a link between PM_10_ concentration and the year.

**Table 3:** Performances of models.

Scenario	Model	Training Sample					Validation Sample					Test Sample				
		RMSE^a^ (µg/m^3^)	R^2,b^ (−)	MAPE^c^ (%)	WIA^d^ (−)	LCI^e^ (−)	RMSE^a^ (µg/m^3^)	R^2,b^ (−)	MAPE^c^ (%)	WIA^d^ (−)	LCI^e^ (−)	RMSE^a^ (µg/m^3^)	R^2,b^ (−)	MAPE^c^ (%)	WIA^d^ (−)	LCI^e^ (−)
Method 1: Multiple Linear Regression (MLR)																
1	PM_10_	7.731	0.183	45.54	0.569	0.137	6.810	0.207	43.66	0.564	0.129	11.657	0.068	28.39	0.554	0.107
2	PM_10_ with PM_10,t-1_	3.551	0.823	19.17	0.811	0.621	3.754	0.811	22.86	0.812	0.623	**1.549**	*0.984*	*5.77*	*0.924*	*0.847*
3	PM_10_ with PM_10,t-2_	3.938	0.778	21.41	0.787	0.574	**4.307**	0.788	27.68	0.783	0.566	**1.579**	*0.982*	*5.42*	*0.924*	*0.847*
4	PM_10_ with PM_10,t-3_	4.317	0.740	23.44	0.769	0.538	**4.395**	0.738	23.62	*0.771*	0.541	**1.670**	*0.979*	*5.96*	*0.916*	*0.831*
5	PM_10,t+1_ with PM_10,t-1_	3.621	0.818	19.12	0.811	0.622	**3.645**	*0.803*	21.94	*0.808*	*0.615*	5.292	0.808	14.80	0.789	0.579
6	PM_10,t+2_ with PM_10,t-1_	4.997	0.651	28.40	0.730	0.460	**4.639**	*0.708*	*23.94*	*0.745*	*0.490*	7.484	0.616	*20.81*	0.699	0.398
7	PM_10,t+3_ with PM_10,t-1_	5.663	0.549	32.30	0.692	0.384	6.143	0.537	27.94	0.702	0.405	8.790	0.473	23.97	0.646	0.292
Method 2: Random Forest (RF)																
1	PM_10_	5.594	0.572	31.12	0.703	0.406	5.710	0.446	30.88	0.667	0.333	10.180	*0.289*	25.64	0.614	0.227
2	PM_10_ with PM_10,t-1_	3.734	0.809	20.09	0.804	0.609	**3.656**	0.775	*20.28*	0.796	0.592	3.371	0.922	8.25	0.878	0.757
3	PM_10_ with PM_10,t-2_	4.173	0.757	22.83	0.780	0.559	4.472	0.724	19.56	0.757	0.514	3.764	0.897	7.15	0.881	0.761
4	PM_10_ with PM_10,t-3_	4.513	0.716	23.94	0.761	0.522	4.583	0.714	*21.81*	0.770	0.540	3.973	0.880	8.44	0.858	0.717
5	PM_10,t+1_ with PM_10,t-1_	3.633	0.818	19.64	0.809	0.618	3.880	0.764	22.09	0.799	0.598	**5.094**	*0.822*	14.85	*0.790*	*0.580*
6	PM_10,t+2_ with PM_10,t-1_	4.933	0.658	27.35	0.733	0.466	5.184	0.653	28.96	0.729	0.458	7.144	*0.651*	21.19	0.699	0.397
7	PM_10,t+3_ with PM_10,t-1_	5.524	0.569	30.23	0.703	0.406	5.986	0.571	30.10	0.714	0.428	8.482	*0.509*	*23.39*	0.664	0.328
Method 3: Extreme Gradient Boosting (XGBoost)																
1	PM_10_	**2.739**	*0.898*	*16.67*	*0.844*	*0.687*	**4.645**	*0.593*	*27.97*	*0.706*	*0.411*	10.428	0.254	*24.89*	*0.616*	*0.232*
2	PM_10_ with PM_10,t-1_	**1.651**	*0.962*	*10.95*	*0.903*	*0.806*	3.675	0.793	20.42	0.794	0.587	4.800	0.842	11.30	0.833	0.666
3	PM_10_ with PM_10,t-2_	**1.846**	*0.953*	*12.26*	*0.891*	*0.782*	4.361	0.691	26.70	0.744	0.487	4.839	0.830	11.68	0.818	0.635
4	PM_10_ with PM_10,t-3_	**2.001**	*0.943*	*13.19*	*0.881*	*0.762*	5.082	0.679	29.93	0.743	0.486	4.189	0.867	11.22	0.823	0.645
5	PM_10,t+1_ with PM_10,t-1_	1.559	0.966	10.64	0.907	0.813	3.924	0.793	*21.30*	0.801	0.602	5.213	0.814	*14.87*	0.782	0.565
6	PM_10,t+2_ with PM_10,t-1_	**2.039**	*0.940*	*14.16*	*0.874*	*0.749*	5.246	0.685	27.89	0.742	0.483	7.840	0.580	22.24	0.679	0.358
7	PM_10,t+3_ with PM_10,t-1_	**2.330**	*0.921*	*16.17*	*0.859*	*0.718*	6.059	0.621	32.79	0.701	0.401	9.456	0.390	24.69	0.634	0.269
Method 4: Artificial Neural Network (ANN)																
1	PM_10_	7.946	0.270	41.95	0.599	0.198	7.192	0.179	40.69	0.559	0.118	**7.612**	0.243	40.20	0.602	0.204
2	PM_10_ with PM_10,t-1_	3.696	0.825	18.92	0.815	0.631	4.366	*0.860*	22.13	*0.817*	*0.635*	3.712	0.808	19.47	0.803	0.606
3	PM_10_ with PM_10,t-2_	4.127	0.789	22.08	0.789	0.577	4.386	*0.803*	*20.54*	*0.806*	*0.612*	4.648	0.757	18.91	0.793	0.586
4	PM_10_ with PM_10,t-3_	4.560	0.752	23.33	0.774	0.549	4.622	*0.798*	22.42	0.789	0.579	3.712	0.766	21.12	0.767	0.535
5	PM_10,t+1_ with PM_10,t-1_	**0.134**	*1.000*	*0.79*	*0.994*	*0.987*	5.563	0.501	31.50	0.707	0.413	5.729	0.592	25.37	0.735	0.471
6	PM_10,t+2_ with PM_10,t-1_	4.939	0.723	27.19	0.747	0.493	5.568	0.521	27.46	0.723	0.446	**5.107**	0.603	24.96	*0.741*	*0.482*
7	PM_10,t+3_ with PM_10,t-1_	5.364	0.648	29.95	0.717	0.435	**5.861**	*0.634*	*28.29*	*0.714*	*0.428*	**6.657**	0.504	32.69	*0.697*	*0.395*

The lowest RMSE values of the model for each scenario (computed on the training, validation and test samples) are in bold. The best results of other statistical performance metrics (computed on the training, validation and test samples) of the model for each scenario are in italics.

^**a**^RMSE = Root mean square error.

^**b**^R^2^ = Determination coefficient.

^**c**^MAPE = Mean absolute percentage error.

^**d**^WIA = Willmott's index of agreement.

^**e**^LCI = Legates and McCabe's index.

The second to fourth scenarios were PM_10_ predictive models with the previous 1, 2 or 3 days’ PM_10_ concentration (PM_10,t-1_, PM_10,t-2_ and PM_10,t-3_, respectively) added to the model. As shown in Table 3, the predictive power of the PM_10_ model in explaining variability tends to increase when the previous day’s PM_10_ concentration (also called the PM_10_ lag effect) was added to the MLR, RF, XGBoost and ANN models. For example, the MLR PM_10_ model with PM_10,t-1_ variable added to the model can explain more variability (by 64% on the training sample, 60% on the validation sample and 92% on the test sample) than that with the time and meteorological parameters only (i.e., Scenario 1). For Scenario 2, this MLR model appears to be a very well fit and acceptably accurate with very good agreement between the predicted and observed daily PM_10_ concentrations with RMSE = 1.549 µg/m^3^, R^2^ = 0.984, MAPE = 5.77%, WIA = 0.924 and LCI = 0.847 during model testing, outperforming the RF, XGBoost and ANN models (Table 3), which was in contrast to previous studies by other researchers^25,26^. This could be due to the differences in location/topography and meteorological conditions of the present study area from those of other studies, resulting in varying dispersal of air pollutants. The variability of PM_10_ of the best MLR model for Scenario 2 was explained by eight interaction variables, which were: (1) (temperature × PM_10,t-1_), (2) (month × year × PM_10,t-1_), (3) (month × wind direction × PM_10,t-1_), (4) (total rainfall × wind direction × PM_10,t-1_), (5) (wind speed × wind direction × PM_10,t-1_), (6) (temperature × wind direction × PM_10,t-1_), (7) (total rainfall × wind speed × wind direction), (8) (total rainfall × wind speed × PM_10,t-1_). The interaction variables (1) to (5) provide significant information in explaining the variability of PM_10_ for Scenario 2.

**Figure 5:**
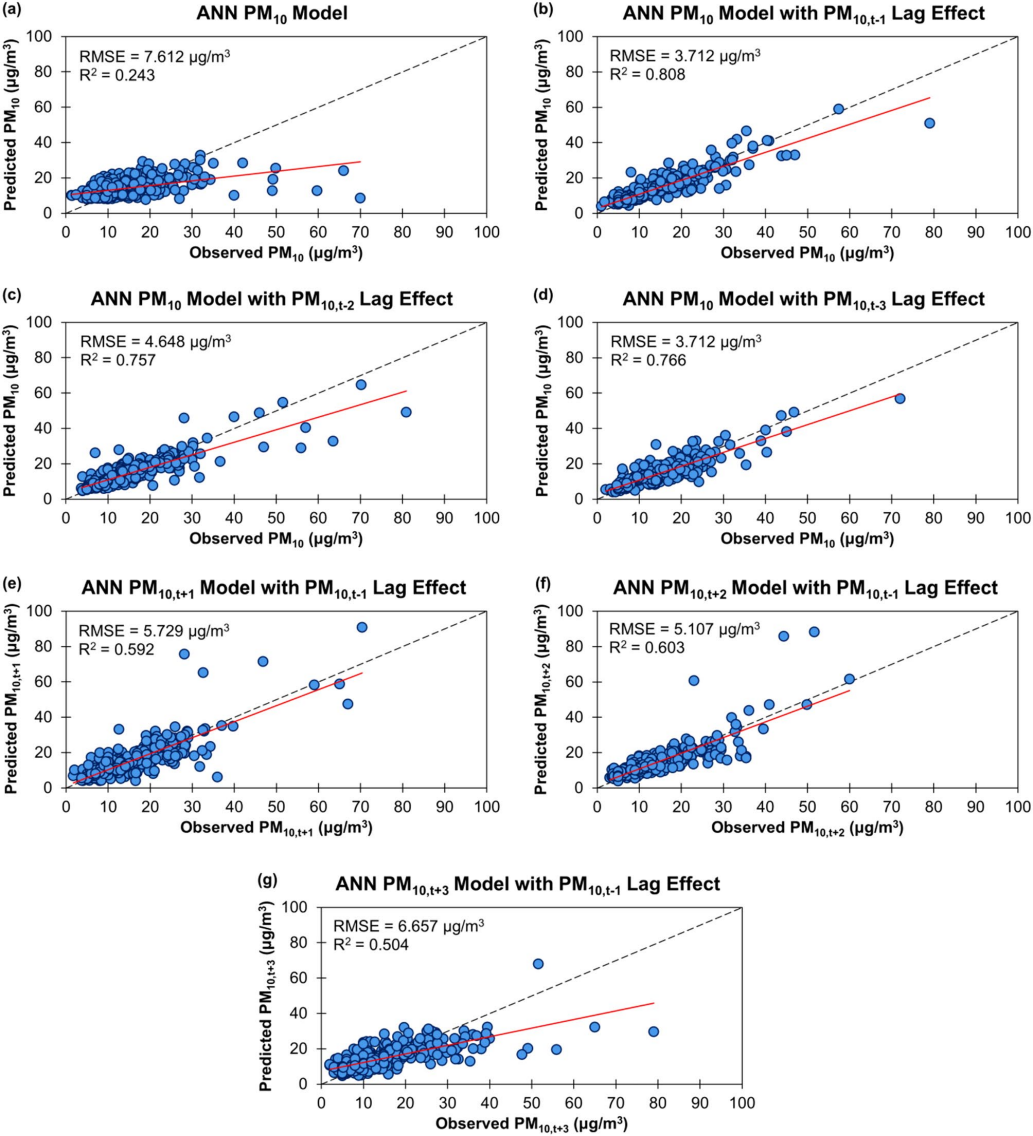
Scatter plots of predicted daily PM_10_ concentrations against observed daily PM_10_ concentrations for the artificial neural network (ANN) PM_10_ models without and with the previous 1, 2 or 3 days’ PM_10_ lag effect (**a–d**), the ANN PM_10,t+1_, PM_10,t+2_ model and PM_10,t+3_ models with the previous day’s PM_10_ lag effect (**e–g**) on the test sample for Brunei-Muara district from 2009 to 2019.

The most influential variable of the PM_10_ models with PM_10,t-1_, PM_10,t-2_ or PM_10,t-3_ variables added to the model was the interaction variables (temperature × PM_10,t-1_), (temperature × PM_10,t-2_) and (temperature × PM_10,t-3_), respectively. Figure 5a–d illustrates the effect of adding PM_10,t-1_, PM_10,t-2_ or PM_10,t-3_ variable to the ANN PM_10_ model during model testing. For all four modelling approaches, it was seen that the models’ performances during training, validation and testing tend to decrease as the number of lag days of the PM_10_ concentration increases (due to increasing errors) and the best model performance was achieved when PM_10,t-1_ variable was added to the model. This indicates that the addition of PM_10,t-1_ variable to the model is sufficient to produce a reliable PM_10_ hindcasting capability.

The fifth and seventh scenarios were PM_10_ predictive forecasting models for the next 1, 2 or 3 days (PM_10,t+1_, PM_10,t+2_ and PM_10,t+3_, respectively) with PM_10,t-1_ variable added to the model. The models’ performances in Table 3 tend to show some reductions in the predictive power of the PM_10_ model in explaining variability for the next 1, 2 or 3 days (PM_10,t+1_, PM_10,t+2_ and PM_10,t+3_, respectively) when compared with the second scenario (i.e., PM_10_ model with PM_10,t-1_ variable added to the model). For example, the MLR PM_10,t+1_ model explain lesser variability (by 0.5% on the training sample, 0.8% on the validation sample and 17.6% on the test sample) than the MLR PM_10_ model with PM_10,t-1_ variable added to the model. The RF PM_10,t+1_ model appears to be a good fit and agreement between the predicted and observed daily PM_10_ concentrations despite having a low but acceptable accuracy during model testing with RMSE = 5.094 µg/m^3^, R^2^ = 0.822, MAPE = 14.85%, WIA = 0.790 and LCI = 0.580, outperforming the MLR, XGBoost and ANN PM_10,t+1_ models (Table 3). This indicates the capability of the random forest models to forecast PM_10_ concentrations for the next day. The most important variable in forecasting the next 2 and 3 days’ PM_10_ concentrations is the PM_10_ variable (with the highest relative contribution of 50% to the RF PM_10,t+1_ model among the variables).

For all four modelling approaches, the predictive power in explaining variability during training, validation and testing decreases as the prediction days were increased (due to increasing errors in the model). The ANN PM_10,t+2_ and PM_10,t+3_ models were considered to have better model performances with a good agreement between the predicted and observed daily PM_10_ concentrations despite having a very low but acceptable accuracy (RMSE = 5.107 µg/m^3^, R^2^ = 0.603, MAPE = 24.96%, WIA = 0.741 and LCI = 0.482 for ANN PM_10,t+2_ model and RMSE = 6.657 µg/m^3^, R^2^ = 0.504, MAPE = 32.69%, WIA = 0.697 and LCI = 0.395 for ANN PM_10,t+3_ model) during model testing than the MLR, RF and XGBoost PM_10,t+2_ and PM_10,t+3_ models (Table 3). The performances of the ANN PM_10,t+1_, PM_10,t+2_ and PM_10,t+3_ models with PM_10,t-1_ variable added to the model during model testing are illustrated in Fig. 5e–g, which show a minor increase (1%) in the explanatory power of the ANN PM_10,t+2_ model and a small decrease (9%) in the explanatory power of the ANN PM_10,t+3_ model when compared to the ANN PM_10,t+1_ models.

## Conclusions

Temporal variations of PM_10_ concentrations and meteorological parameters (that include the daily temperature, wind speed, wind direction and total rainfall) in Brunei-Muara district from 2009 to 2019 were examined in this study. The Pearson correlation analysis showed that PM_10_ increases with increasing atmospheric temperature. More PM_10_ can be blown away if the wind speed increases and the wind blows from the NNE direction. Heavier rain can also wash away more PM_10_ in the atmosphere, thus improving the air quality in the studied area. Observations on the combined effects of rain and wind conditions in Brunei-Muara district from 2009 to 2019 revealed that moderate rain with light air reduced the most PM_10_ pollution in the area, with a 7% reduction in the mean daily PM_10_ concentrations from the previous day’s PM_10_ concentration. The MLR PM_10_ models, particularly with the previous day’s PM_10_ lag effect (PM_10,t-1_), can be used to predict daily PM_10_ concentrations more accurately than the RF, XGBoost and ANN PM_10_ models, provided that the meteorological conditions are known. Meanwhile, the RF PM_10,t+1_ model with PM_10,t-1_ variable added to the model showed more accurate forecasts for the next day’s PM_10_ concentration and the ANN PM_10,t+1_ model with PM_10,t-1_ variable added to the model showed more accurate forecasts for the next 2 and 3 days’ PM_10_ concentrations. This research can provide a method for predicting PM_10_ concentrations for the studied area where PM_10_ concentration data are not available. Due to the rapid climate change, it was recommended to improve the PM_10_ predictive forecasting models’ ability to capture greater daily variability of PM_10_ through the inclusion of meteorological and/or PM_10_ concentrations data in Brunei-Muara district beyond 2018 on the models in future studies when available.

## Data Availability

The data that support the findings of this study are available from the Brunei Darussalam Meteorological Department (BDMD), Ministry of Transport and Infocommunications, Brunei Darussalam and the Department of Environment, Parks and Recreation (JASTRe), Ministry of Development, Brunei Darussalam but restrictions apply to the availability of these data, which were used under license for the current study, and so are not publicly available. Data are however available from the corresponding author (W.Y. Hong) upon reasonable request and with permission of BDMD and JASTRe, Brunei Darussalam.
